# A Sustainable Alternative to PVDF for Neural Tissue Engineering via Piezoelectric PHBV and Cellulose Acetate Fibers

**DOI:** 10.1021/acsbiomaterials.5c01876

**Published:** 2026-02-02

**Authors:** Joanna E. Karbowniczek, Pelin İlhan, Piotr K. Szewczyk, Ecenaz Merve Namli, Martyna Polak, Zehra Gül Morçimen, Joanna Knapczyk-Korczak, Ezgi Turunç, Aylin Şendemir, Urszula Stachewicz

**Affiliations:** † Faculty of Metals Engineering and Industrial Computer Science, AGH University of Krakow, Al. A. Mickiewicza 30, 30-059 Kraków, Poland; ‡ 37509 Ege University, Faculty of Engineering, Bioengineering Department, Biomaterials and 3D Biointerphases Laboratory, 35100 Izmir, Türkiye; § Ege University, Institute of Health Sciences, Stem Cell Department, 35100 Izmir, Türkiye; ∥ Katip Çelebi University, Faculty of Pharmacy, Department of Basic Pharmaceutical Sciences, 35100 Izmir, Türkiye; ⊥ Ege University, School of Natural and Applied Sciences, Biomedical Technologies Department, 35100 Izmir, Türkiye

**Keywords:** Piezoelectric scaffolds, Electrospun aligned fibers, PHBV, Neural tissue engineering, Calcium signaling, Neurite outgrowth

## Abstract

Poly­(vinylidene fluoride) (PVDF) is widely used in neural tissue engineering for its strong piezoelectric response, yet its nonbiodegradability and environmental persistence limit its clinical translation. Neural regeneration demands scaffolds that not only replicate the extracellular matrix but also deliver bioelectrical cues to guide neuronal growth. Here, we introduce aligned electrospun fibers of poly­(3-hydroxybutyrate-*co*-3-hydroxyvalerate) (PHBV) and cellulose acetate (CA) as biodegradable, sustainable alternatives to PVDF for studying how piezoelectricity, surface charge, and nanotopography influence neuronal function. Compared to polycaprolactone (PCL) as a nonpiezoelectric control, the PVDF, PHBV, and CA scaffolds exhibited distinct morphologies and progressively decreasing piezoelectric coefficients. All supported robust adhesion and proliferation of B35 neuronal cells; however, piezoelectric fibers significantly enhanced intracellular Ca^2+^ influx, neurite elongation, and β3-tubulin expression. Both PVDF and PHBV activated the WNT/GSK3β signaling pathway and downregulated the pro-apoptotic BAX/BCL-2 ratio, suggesting enhanced neuroprotective capacity. Notably, while PVDF induced strong Ca^2+^-mediated neuronal maturation through piezoelectric stimulation, PHBV elicited additional antiapoptotic effects, likely linked to 3-hydroxybutyrate metabolism. Together, these findings demonstrate that combining nanoscale alignment, surface charge, and intrinsic piezoelectricity generates a bioelectrically active microenvironment conducive to neuronal regeneration. Importantly, PHBV emerges as a sustainable, biodegradable alternative to PVDF, bridging environmental responsibility with functional performance in neural tissue engineering.

## Graphical abstract

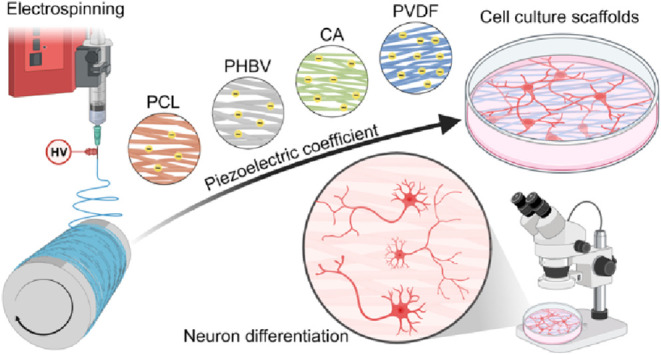

## 1 Introduction

Neural tissue regeneration, particularly in the context of spinal cord injury (SCI), remains one of the most significant challenges in regenerative medicine. Global estimates suggest that approximately 15 million people are living with SCI as a result of trauma, accidents, violence, tumors, degenerative and vascular conditions, infections, toxins, or birth defects.^1^ SCI often leads to permanent neurological deficits with varying degrees of paralysis, due to the limited regenerative capacity of the central nervous tissue.^2^ To overcome these limitations, tissue-engineered scaffolds have emerged as promising tools to guide axonal regrowth, provide mechanical support, and stimulate cellular responses necessary for regeneration.^3^ Among the many factors influencing scaffold performance, morphology,^4,5^ surface properties,^6,7^ and piezoelectricity^8^ have been identified as critical parameters for promoting neural cell adhesion, neurite outgrowth, and functional recovery. The morphology of scaffolds, particularly fiber diameter, can influence cellular behavior, including attachment, spreading, and ingrowth. Microfibers facilitate cell migration within the scaffold structure^9^ and promote cellular elongation.^10^ Aligned fibers are known for enhancing directional nerve cell growth.^4,5,11,12^ Moreover, fibers with diameters exceeding 0.75 μm were shown to improve neurite extensions along the fibers effectively,^4^ which can be further improved by nanopatterning of the fibers’ surface in the form of grooved morphology.^13^

Electrospun polymer scaffolds have gained significant attention in neural tissue engineering because they closely replicate the fibrous structure of the extracellular matrix (ECM), offer physical guidance for axonal alignment, and function as effective platforms for bioelectrical stimulation.^4,14^ In respect to the central nervous system, including brain tissue, biomaterials possessing piezoelectric and conductive properties are of high importance to support tissue regeneration as well as to design a variety of probing and monitoring electrodes.^15,16^ Numerous polymers have been electrospun into fibrous scaffolds for neural applications, including poly­(vinylidene fluoride) (PVDF),^17^ poly­(3-hydroxybutyrate-*co*-3-hydroxyvalerate) (PHBV),^18^ cellulose acetate (CA),^19^ and polycaprolactone (PCL).^20^ PVDF is widely recognized as one of the most piezoelectric polymers, capable of generating electrical stimuli in response to mechanical deformation,^21^ thereby potentially activating voltage-gated calcium channels and enhancing neural activity. However, its production and use are increasingly restricted in the European Union due to environmental and regulatory concerns,^22^ necessitating the search for alternative piezoresponsive polymers with lower environmental impact but sufficient functionality for neural scaffold applications. The planned restrictions regarding PVDF usage are related to its classification among per- and polyfluorinated alkyl substances (PFAS), demonstrating adverse health effects as well as a high level of bioaccumulation and pollution in water, soil, and other environments. In many studies related to polymeric materials’ life cycle assessment (LCA), evaluating the overall environmental impact of materials throughout their manufacturing, use, and final disposal is gaining more and more attention.^23^ While selecting proper biomaterials for medical applications, we should also consider the principles of LCA; however, we cannot forget the primary goal related to biocompatibility and patients’ safety.

PHBV, a biocompatible and biodegradable copolymer derived from microbial fermentation, offers mild piezoelectricity and has been explored for soft tissue engineering,^24^ though its neural-specific applications remain under-investigated. Similarly, CA, a polysaccharide-derived polymer, combines good cytocompatibility with tunable mechanical properties and has been electrospun into scaffolds supporting various cell types,^25^ including neuronal cells.^26^ Both PHBV and CA represent potentially safer alternatives to PVDF, offering moderate piezoelectric activity that could still stimulate neural cells. In contrast, PCL is a well-known nonpiezoelectric polymer commonly used as a control material in neural studies because of its stable morphology and excellent biocompatibility, despite its limited bioelectrical activity.^20^ Previous studies have shown that electrospun fibers from these polymers can support neural cell adhesion, proliferation, and neurite extension. Still, systematic comparisons of their piezoelectric properties and the resulting biological responses remain scarce. Particularly, the relationship between fiber piezoelectricity, surface charge, and intracellular calcium signaling, a key factor in neurite extension and synaptic activity, has not been fully elucidated.

In this study, we aimed to compare piezoelectric polymers, PVDF, PHBV, and CA, in the form of aligned electrospun fiber scaffolds and assess their effects on neurite extension along the fibers, intracellular calcium levels, and expression of specific marker genes in neural processes (including metabolic regulation, microtubule dynamics, cell signaling, and apoptosis). These materials were benchmarked against nonpiezoelectric aligned PCL fibers. The fiber morphology and diameters were characterized by scanning electron microscopy (SEM), the chemical composition was evaluated using Fourier transform infrared spectroscopy (FTIR), and wettability was determined via water contact angle measurements. Furthermore, the surface charge of each scaffold was quantified through streaming ζ-potential measurements at physiological pH, and its piezoelectric coefficients were assessed with a d_33_ meter. For biological assessment, neuronal cell line B35 was cultured on the electrospun fibers, and cell morphology was evaluated using confocal laser scanning microscopy (CLSM) and SEM. A calcium sensitivity dye (Fluo-4 NW) was used to study intracellular calcium levels, and qPCR analysis was used to compare the tested gene expressions. The summarized workflow within this research is presented in Scheme 1. We hypothesize that piezoelectric scaffolds can provide bioelectrical stimulation to neural cells, activating voltage-gated calcium channels, thereby increasing intracellular calcium levels and promoting neurite outgrowth. By identifying viable alternatives to PVDF with sufficient piezoelectric activity and cytocompatibility, this work seeks to advance the development of next-generation neural scaffolds with improved safety, functionality, and regulatory compliance.

**Scheme 1:**
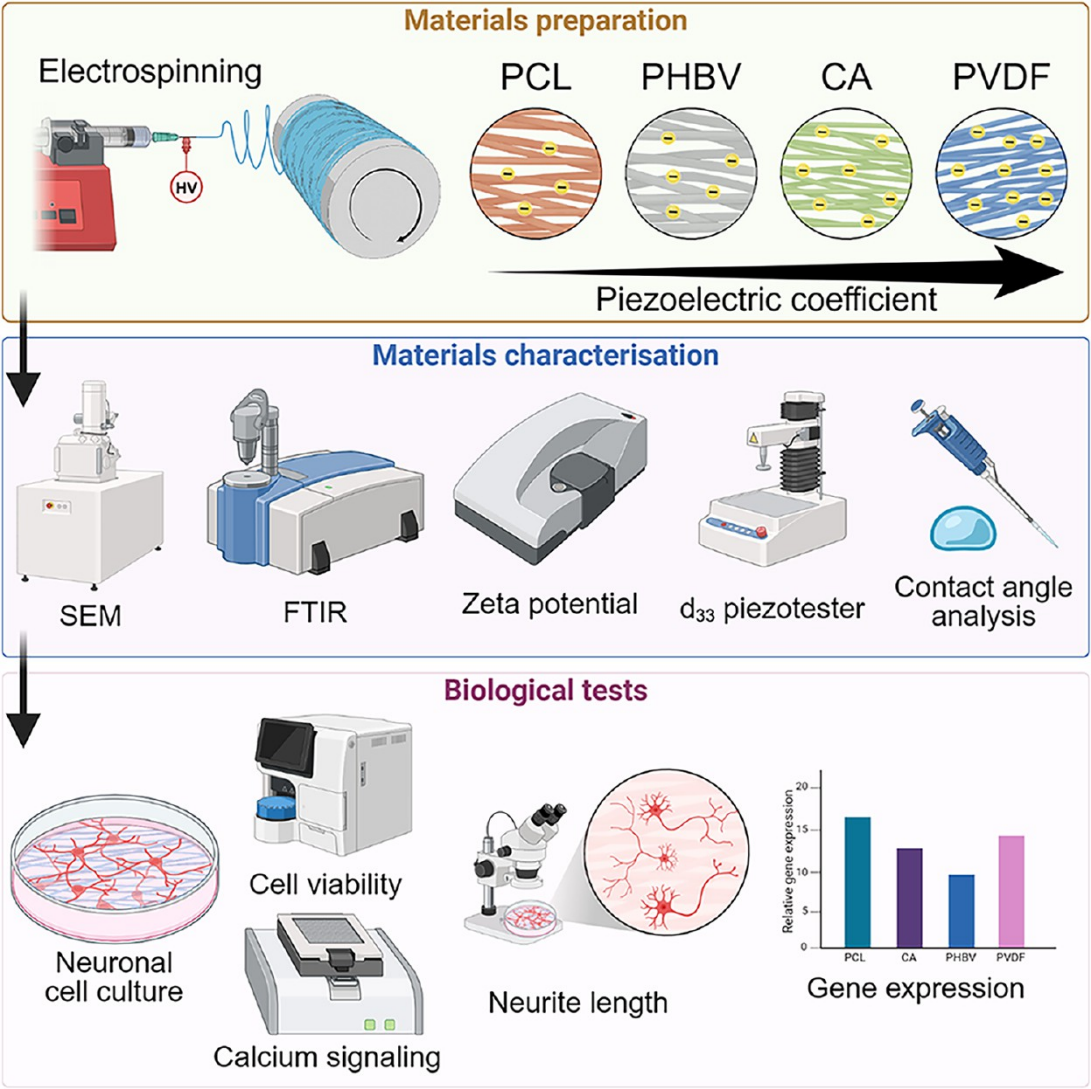
Graphical Representation of the Workflow Within This Research, Underlining Materials Manufacturing by Electrospinning, Along With Analytical Methods Used for Aligned Fibers Characterization and Extensive Biological Investigation

## 2 Materials and Methods

### 2.1 Electrospinning of Aligned Fibers

For this study, four types of aligned electrospun fibers from different polymers were produced using an electrospinning setup equipped with a climate controlled chamber (IME, The Netherlands). To prepare solutions, polymers were dissolved in solvent mixtures using a magnetic stirrer with a heating plate (IKA, Germany). Polyvinylidene fluoride (PVDF, Sigma-Aldrich, USA) solution in a concentration of 24 wt % was prepared by dissolving the polymer in solvents dimethylacetamide (DMAC, Avantor, Poland): acetone (1:1 v/v) by mixing for 3 h at *T* = 60 °C. Poly­(3-hydroxybutyrate-*co*-3-hydroxyvalerate) PHBV powder (PHV content 2 wt %, *M*_w_ = 450 000 g·mol^–1^, Helian Polymers, The Netherlands) was dissolved in chloroform: dimethylformamide (DMF, Avantor, Poland) (9:1 v/v) by mixing for 4 h at *T* = 45 °C to achieve a concentration of 10 wt %. Cellulose acetate (CA, average *M*_w_ = 50,000 g·mol^−1^, Sigma-Aldrich, USA) in the concentration of 19 wt % was dissolved in DMAC: acetone (1:1 v/v) by mixing for 5 h at *T* = 25 °C. Polycaprolactone (PCL, *M*_w_ = 80,000 g·mol^–1^, Sigma-Aldrich, USA) was dissolved in a concentration of 8 wt % in chloromethane: methanol (7:3 v/v). Details of the electrospinning parameters for each solution are listed in Table 1. To achieve an oriented distribution of fibers, the rotation speed of the collector for each polymer solution was set above 1500 rpm. Fibers were collected onto baking paper to facilitate their removal for further tests.

**Table 1:** Summary of the Electrospinning Parameters Used to Prepare Aligned Fibers of PVDF, PHBV, CA, and PCL

parameter/polymers	PVDF	PHBV	CA	PCL
concentration [%]	24	10	19	8
Solvents	DMAC: acetone 1:1	chloroform: DMF 9:1	DMAC: acetone 1:1	chloromethane: methanol 7:3
voltage [kV]	15	17	15	16
flow rate [mlh^–1^]	1.2	6	0.1	3.5
distance [cm]	11	20	20	16
collection time [min]	25	40	240	30
rotation speed [rpm]	1500	2500	2000	2500
temperature [°C]	25	25	25	30
humidity [%]	60	40	70	30

### 2.2 Fibers’ Morphology and Chemical Composition

The morphology of prepared PVDF, PHBV, CA, and PCL electrospun scaffolds was evaluated by SEM (Merlin, Zeiss, Germany) with an applied voltage of 2 kV, a current of 100 pA, and a working distance (WD) below 8 mm, using a secondary electron (SE) detector. Prior to imaging, samples were coated with an 8 nm layer of Au by a rotary pump sputter coater (Q150RS, Quorum Technologies, United Kingdom). The average fiber diameter for each scaffold was calculated based on 100 measurements of fibers from several SEM images per sample using ImageJ software (version 1.53, USA). Additionally, fibers’ orientation was analyzed using ImageJ software with a Directionality plugin. Chemical composition of fibers was evaluated by attenuated total reflectance Fourier transform infrared spectroscopy (ATR-FTIR) using a Nicolet iS 5 FTIR Spectrometer (Thermo Fisher Scientific, USA) with the diamond crystal. For each sample, 64 scans with a resolution of 4 cm^–1^ were recorded between 2000 and 400 cm^–1^.

### 2.3 Wettability and ζ-Potential

The water contact angle of aligned PVDF, PHBV, CA, and PCL fibers was measured to evaluate their wettability. Droplets of deionized water (Spring 5UV purification system, Hydrolab, Poland) measuring 3 μL were applied onto the surface of each material using a pipette, and the images were recorded with a Canon EOS 700D camera and an EF-S 60 mm f/2.8 Macro USM zoom lens. The average water contact angle was obtained from measurements of five droplets per sample by using ImageJ software.

Streaming ζ-potential for PVDF, PHBV, CA, and PCL aligned fibers was measured with an electrokinetic analyzer for solid surfaces (SurPASS 3, Anton Paar, Austria) with a cylindrical cell dedicated to porous materials. Four measurements of each material type were done in the 0.01 M KCl solution at pH = 7 adjusted by adding 0.05 M NaOH (Avantor, Poland). The constant pH value was selected as the representation of *in vitro* conditions in the cell culture medium. The permeability index during electrolyte flow was approximately 140 for each measurement.

### 2.4 Piezoelectric Coefficient Measurement

The piezoelectric coefficients of all the samples were assessed by utilizing a d_33_ meter (YE2730A d_33_ meter, Sinocera, China). Before testing, a 6 nm Au80–20Pd layer was deposited as electrodes on both sides of the fiber mats. Each sample was measured 10 times in different locations to calculate the average d_33_ value. A reference sample, a low-d_33_ (2.2 pCN^–1^) piezoelectric single crystal from PolyK (USA), was used for instrument calibration.

### 2.5 Neural Cell Culture and Cell Viability on Piezoelectric Fibers

The rat neuronal cell line (B35) was cultured in a medium containing Dulbecco’s Modified Eagle Medium/Nutrient Mixture F12 (DMEM F12), 10% fetal bovine serum (FBS), 1% l-glutamine, and 0.1% gentamycin. PVDF, PHBV, CA, and PCL electrospun fibers were cut into 1 × 1 cm^2^ pieces, placed in Cell Crowns inserts (Scaffdex, Finland), and sterilized using ethylene oxide. The scaffolds were aerated for 3 days, washed with phosphate-buffered saline (PBS), and conditioned overnight in serum-containing DMEM F12 medium. Cells were seeded onto the electrospun fibers dropwise at a concentration of 2 × 10^4^ cells/cm^2^ to perform viability analysis on days 1, 3, and 7, and for each time point, three samples of each material type were used.

Alamar Blue assay was used for the viability analysis. Alamar Blue dye is a resazurin-based solution used to quantitatively measure the viability. After resazurin diffuses into live cells, it is reduced to resorufin, a red fluorescent compound. Thus, changes in viability can be detected by using absorbance or fluorescence-based plate readers. After adding serum-free medium containing 10% Alamar Blue to the samples, they were incubated in a CO_2_ incubator at 37 °C for 4 h. Then, the media containing resorufin was read in a spectrophotometer (Synergy HTX Multi-Mode Microplate Reader, BioTek Instruments) at 570/600 wavelengths.

### 2.6 Intracellular Calcium Response

Cells were seeded onto the fibers, as described. Amlodipine (1000 μM) was used as a Ca^2+^ blocker. Amlodipine blocks voltage-dependent L-type calcium channels, thus preventing calcium influx.^19^ For kinetic measurements, KCl was used for the depolarization of neurons. The media were changed every 2 days, and the cells were cultured for 7 days. Kinetic Ca^2+^ assay was performed with Fluo-4 NW Calcium Assay Kit (Invitrogen-F36206) on the first, third, and seventh day of culture. On the day of the experiment, cells were washed twice with PBS and incubated at 37 °C for 30 min. Then at room temperature for an additional 30 min with the cell-permeant fluorescent Ca^2+^ indicator, Fluo-4 NW (2.5 M), in Hank’s balanced salt solution (HBSS) containing 20 mM 4-(2-hydroxyethyl)-1-piperazineethanesulfonic acid (HEPES and 2.5 mM probenecid). For kinetic measurements, 50 mM KCl was added to the cells, and fluorescence was recorded immediately at 1 min intervals for a total of 10 min. For the recordings, the temperature was maintained at 37 °C. Fluorescence was excited at 485/20 nm, and emission was measured at 528/20 nm in a multidetection microplate reader (Synergy HTX Multi-Mode Microplate Reader, BioTek Instruments). Four samples of each material type were tested at each time point.

### 2.7 Imaging and Neurite Analysis

After 7 days of cell growth on the fibers, the samples were prepared for immunofluorescent staining. The fibers were placed in a separate culture dish and washed with PBS containing Ca^2+^ and Mg^2+^. Cells were fixed in cold 4% paraformaldehyde for 30 min. Sudan Black dye was applied prior to immunofluorescence staining to reduce the autofluorescence of the fibers. The dye was prepared in 70% ethanol, applied to all samples, and incubated at room temperature for 4 h. The samples were then washed three times with a 0.2% Triton X-100 solution at room temperature. To block nonspecific binding of immunofluorescent dyes, 1% bovine serum albumin (BSA) prepared in PBS was applied. Antibeta 3-Tubulin primary antibody solution at a concentration of 1 μg/mL was prepared in 1% BSA-PBS. After overnight incubation at 4 °C, the samples were washed with 0.2% PBS-Triton X-100 solution on a shaker. A secondary antibody solution containing anti-rabbit antibodies conjugated with Alexa Fluor 647 (Abcam ab150075; 1:500) and DAPI (4′,6-diamidino-2-phenylindole, Dihydrochloride-Sigma; 1:1000) was prepared in 1% BSA-PBS. They were incubated at 37 °C for 2 h. After being washed in PBS, the samples were transferred onto a slide, covered with Mowiol 4-88 (Sigma-Aldrich, USA), and observed under a fluorescence microscope (Leica DM4000). Neurite lengths were analyzed with ImageJ software (version 1.53, USA).^27^ At least 6 measurements were taken from at least 3 photographs from each group. GraphPad Prism (GraphPad Software, Boston, Massachusetts, USA, www.graphpad.com) was used for statistical analysis.

Additional samples were prepared for morphology imaging by SEM. Fixed cells after 7 days of incubation on aligned electrospun scaffolds were dehydrated in a series of ethanol solutions (50, 70, 80, 90, 100%—10 min incubation in each). Subsequently, samples were placed on aluminum stubs and sputter-coated with an 8 nm layer of Au and imaged by SEM with settings previously described for fibers’ morphology.

### 2.8 Gene Expression Analysis

The quantitative PCR (qPCR) analysis of the expression of the following genes: BCL-2, Bax, β3-tubulin (Tubb3), GSK3B, and Wnt1 was performed through mRNA quantification on three samples of PVDF, PHBV, CA, and PCL fibers. These genes were selected for their roles in cell differentiation, apoptosis regulation, cytoskeletal dynamics, and signaling pathways, such as Wnt/β-catenin. Piezoelectric effects, which influence mechanical stress and charge distribution within cells, can alter the expression of these genes, leading to changes in cell proliferation, differentiation, and survival. Specifically, the piezoelectric properties of the fibers could modulate key cellular processes such as microtubule dynamics (via Tubb3), apoptosis (through Bcl-2 and Bax), metabolic regulation (GSK3B), and cell signaling (Wnt1), making them crucial targets for understanding how mechanical forces impact cellular function and tissue development. The primer sequences for the target genes and the housekeeping gene β-actin (Actβ) were designed based on the gene regions obtained from the National Center for Biotechnology Information (NCBI) GenBank. Using the cDNA sequences and conserved regions of these genes, primers, and probes were optimized with ClustalW alignment and Oligo7 software. The specificity of the designed primers and probes was verified by using the BLAST program.

For all experimental groups, the media were removed from the fibers, and the samples were washed with Ca^2+^/Mg^2+^-free PBS. Subsequently, cells were detached from the fibers using trypsin and transferred to 2 mL Eppendorf tubes. Cells were centrifuged at 1000 rpm for 5 min and lysed using the cell lysis solution provided in the MasterPure DNA and RNA Purification Kit (Bioresearch Technologies, MC85200). RNA isolation was performed according to the manufacturer’s protocol, which included enzymatic treatment with DNase to eliminate DNA contamination, followed by purification steps to ensure high RNA quality. The RNA was quantified spectrophotometrically, and the A260:A280 ratio was measured to confirm purity. RNA samples were stored at −86 °C until further use.

The samples were then incubated in a thermal cycler at 70 °C for 5 min to denature the RNA. After the samples were removed from the thermal cycler, 12 μL of the master mix (containing thermostable DNA polymerase, dNTPs (deoxynucleotide triphosphates), and buffer) was added to each sample. The tubes were placed back in the thermal cycler and processed under the following conditions: 25 °C for 5 min, 42 °C for 60 min to allow reverse transcription, and 80 °C for 5 min to inactivate the reverse transcriptase. The cDNA samples were stored at −20 °C for future use.

To quantify the PCR products of target genes, single-stranded cDNA was amplified by using sequence-specific primers. The cDNA samples were analyzed for gene expression using the Stratagene Mx3000P device with the primer sets listed in Table 2. The qPCR reaction was performed following the protocol below: an initial denaturation step at 95 °C for 3 min, followed by 40 cycles of denaturation at 94 °C for 20 s, primer annealing at 60 °C for 20 s, and extension at 95 °C for 1 min, 55 °C for 30 s, and 95 °C for 3 s. The reaction program was designed to allow for the amplification and quantification of the target genes. Gene expression levels were calculated using the 2^–ΔΔCt^ method.

**Table 2:** Primers Used, Along With Their Respective NCBI Gene IDs and Forward/Reverse Primer Sequences

NCBI Gene ID	primers
RBcl-2 (NM_016993.2)	Forward: 5′- GAGTACCTG AACCGGCATCT −3′Reverse: 5′- GAAATCAAACAGAGGTCGCA −3
RBax (NM_017059.2)	Forward: 5′-CCAGGATCGAGCAGAGAGGA-3′ Reverse: 5′- TGTTGTCCAGTTCATCGCCA-3′
RTubb3 (NM_139254.2)	Forward: 5′- CCTGCCTCTTCGTCTCTAGC −3′ Reverse: 5′- TCCCAGAACTTGGCCCCTAT −3′
RGSK3B (NM_032080.1)	Forward: 5′-CTGGCCACCATCCTTATCCC-3′ Reverse: 5′- AGAAGCGGCGTTATTGGTCT-3′
RWnt1 (NM_001105714.1)	Forward: 5′-AATCCTGCACCTGCGACTAC-3′ Reverse: 5′-CAGAGTCCACGAACTCTCGG-3′

### 2.9 Statistical Analysis

All the results presented in this study are shown as mean ± SD (standard deviation), and at least three independent repeats were conducted for each experiment. Statistical analysis was performed by one-way ANOVA, followed by Tukey’s post hoc test. Statistically significant differences between groups were marked at several significance levels: *p < 0.05, **p < 0.01, ***p < 0.001.

## 3 Results

### 3.1 Morphology and Properties of Electrospun Fibers

To verify neural cell interactions with polymer scaffolds and their eligibility for tissue engineering, we electrospun aligned fibers of PVDF, PHBV, CA, and PCL, and their morphologies are presented in Figure 1a–d. CA and PCL fibers were smooth with average fiber diameters of 0.50 ± 0.20 and 0.67 ± 0.45 μm, respectively. While PVDF fibers had a grooved morphology and average fiber diameter of 0.94 ± 0.39 μm, PHBV fibers exhibited surface porosity and a significantly higher average fiber diameter of 2.07 ± 0.45 μm. Measured values of fiber diameters are presented as a histogram in Figure 1e. Fibers’ directionality measurements show a very high alignment level, especially for CA and PHBV meshes, achieved by high rotation speed of the collector during manufacturing (Figure 1f). For CA fibers, over 15% were perfectly aligned in a single direction, with all fibers oriented within 20° of the reference axis. In contrast, PHBV fibers showed nearly 10% perfect alignment, with the remaining fibers distributed within 30° of the reference axis. In the case of PVDF and PCL fibers, the degree of alignment was less pronounced, with fewer than 5% of fibers being perfectly oriented and some deviating up to 40° from the reference axis. Moreover, in these less aligned samples, fibers stretched by the electric field during electrospinning appeared to relax upon reaching the collector, resulting in a wavy morphology that reduced their overall directionality. Such wavy fiber morphology is usually achieved by magnetic-field-assisted electrospinning or increased flow rate of polymer solution^28^ and can be beneficial for the growth of musculoskeletal interfacial tissues.^29^ The interplay of various forces during the electrospinning process: electrostatic, mechanical stretching, surface tension, gravity, and air flow is difficult to balance perfectly and they are causing the whipping motion of the polymer jet and decreasing the level of fiber alignment.^29^ Therefore, some of the manufactured fibers, like PVDF or PCL, are not ideally ordered despite applying a high rotation speed of the collector.

**Figure 1:**
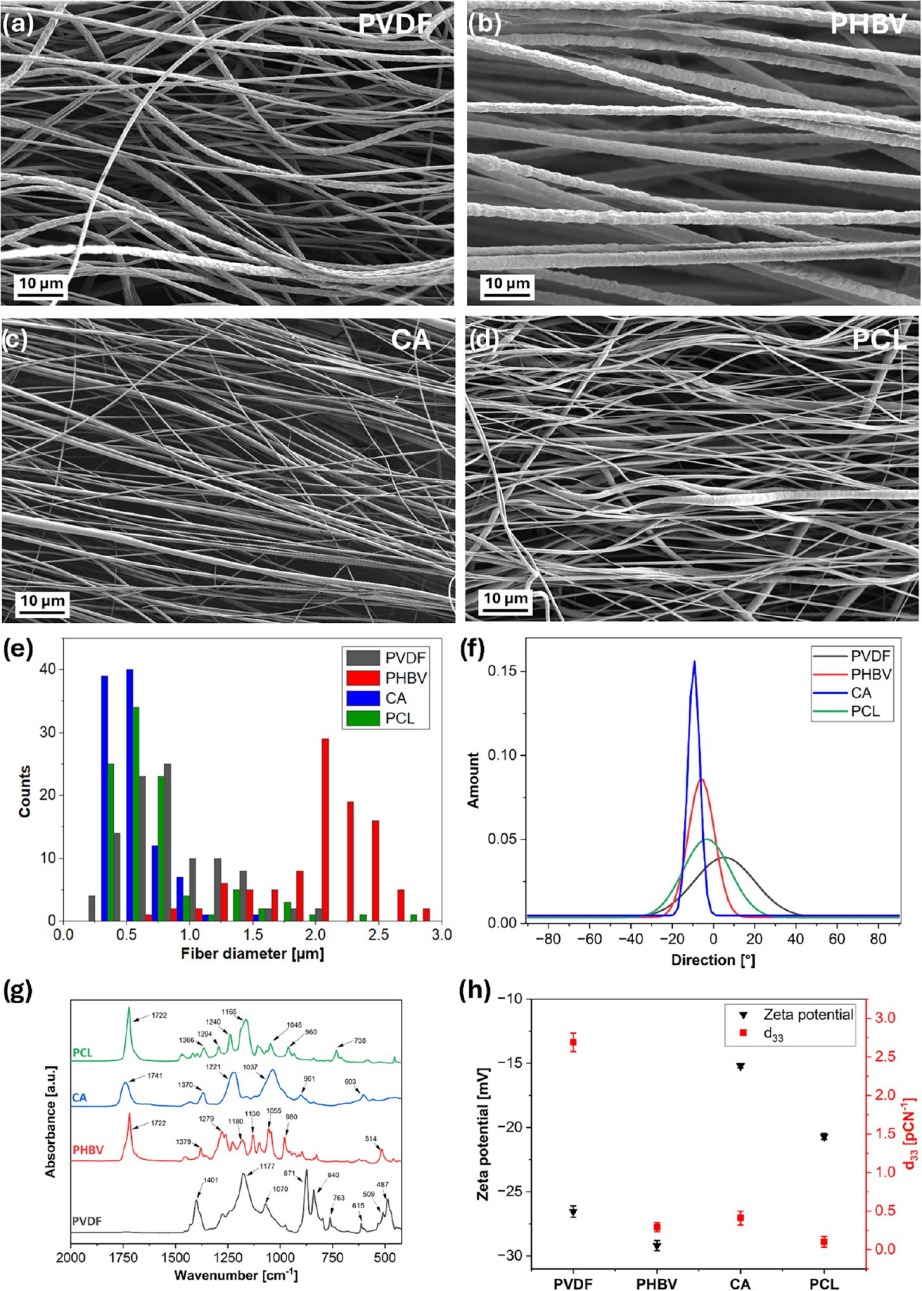
Structural and physicochemical characterization of aligned electrospun fibers. SEM micrographs of aligned electrospun fibers: (a) PVDF, (b) PHBV, (c) CA, (d) PCL, (e) histogram showing the distribution of fiber diameters, (f) fiber directionality (Gaussian fit), (g) FTIR spectra of all electrospun scaffolds, and (h) ζ-potential and piezoelectric coefficient (d_33_).

FTIR was performed to identify the chemical composition and confirm the presence of functional groups on the electrospun fibers. The analysis presented in Figure 1g revealed all typical peak characteristics of electrospun polymers. Carbonyl group C=O stretching was recorded at 1722 cm^–1^ for both PCL and PHBV and at 1741 cm^–1^ for CA, similar to what was described in the literature.^25,30,31^ Additionally, for those polymers, various aliphatic C–H vibrational bands were observed in the range of 1220–1470 cm^–1^ and 900–980 cm^–1^, while C–O vibrational bands were recorded in the range 1050–1200 cm^–1^. For PVDF, specific bands corresponding to the β phase responsible for its piezoelectric properties were identified at 509 cm^–1^ (CF_2_ bending) and 840 cm^–1^ (CH_2_ rocking), along with bands characteristic of the α phase at 763 cm^–1^, associated with CF_2_ bending vibrations.^32^

Water contact angle measurements were carried out to evaluate the wettability of the electrospun scaffolds. Aligned PVDF, PHBV, CA, and PCL electrospun fibers exhibited a strongly hydrophobic nature, with water contact angles: 122.3 ± 6.9°, 117.3 ± 4.8°, 113.2 ± 4.6°, and 114.4 ± 4.2° for PVDF, PHBV, CA, and PCL, respectively. The measured values correlate well with those reported in the literature for contact angles of electrospun fibers.^25,33–35^

ζ-Potential measurements of the aligned electrospun scaffolds revealed negative surface charges for all tested samples: −26.54 ± 0.44, −29.20 ± 0.39, −15.19 ± 0.16, and −20.70 ± 0.23 mV for PVDF, PHBV, CA, and PCL, respectively (Figure 1h). Interestingly, in the case of PHBV and CA fibers, electrospinning with high rotation speed, allowing fiber orientation, resulted in more negatively charged surfaces compared to random fibers. Previously reported ζ-potential values were approximately −18 mV^36^ and −12 mV^25^ for PHBV and CA random fibers, respectively.

Piezoelectric measurements were performed to evaluate the ability of the electrospun scaffolds to generate electrical signals in response to mechanical deformation, a property known to stimulate neuronal activity and support tissue regeneration.^8,37,38^ The piezoelectric response was assessed using calibrated quasi-static d_33_ measurements, which, for thin and compliant fibrous mats, should be interpreted as effective relative indicators rather than intrinsic material constants. Our analysis reveals clear differences in the piezoelectric responses across samples (Figure 1h). PVDF exhibited the highest effective piezoelectric coefficient of 2.69 ± 0.12 pCN^–1^, consistent with its well-established strong piezoelectricity.^39^ In contrast, PHBV and CA showed lower responses of 0.29 ± 0.06 pCN^–1^ and 0.41 ± 0.09 pCN^–1^, falling within the ranges reported for electrospun fibrous systems in the literature. To ensure reliable measurements in the low-signal regime, the d_33_ meter was calibrated using a low-d_33_ PVDF film reference, while polycaprolactone (PCL) served as a nonpiezoelectric negative control and^39,40^ produced a negligible signal (0.10 ± 0.047pCN^–1^), within the margin of experimental error.^40^ Under these controlled conditions, the measurements enable a meaningful comparative assessment of the piezoelectric response of the different electrospun scaffolds.

### 3.2 Neuronal Cell Responses to Electrospun Scaffolds

To evaluate the biocompatibility and functional response of neuronal cells to the electrospun scaffolds, proliferation and Ca signaling studies were performed using the B35 cell line. As expected, the number of B35 cells increased with longer incubation times on the aligned scaffolds. After 7 days of cell growth, the highest cell viability was measured on PVDF fibers, where aligned morphology and piezoelectric properties created a beneficial microenvironment for neural cell growth (Figure 2a). Fluo-4 AM is a widely used fluorescent indicator for measuring intracellular calcium signaling, which can be triggered by agonists and inhibited by antagonists. Amlodipine, a calcium channel blocker that works by inhibiting voltage-gated L-type Ca^2+^ channels, was used to prevent the Ca^2+^ influx. As the goal of the piezoelectric fibers was to enhance Ca^2+^ release through external stimuli, it was demonstrated that Ca^2+^ release characteristics are different when the Ca^2+^ channels of the cells are active and blocked.

**Figure 2:**
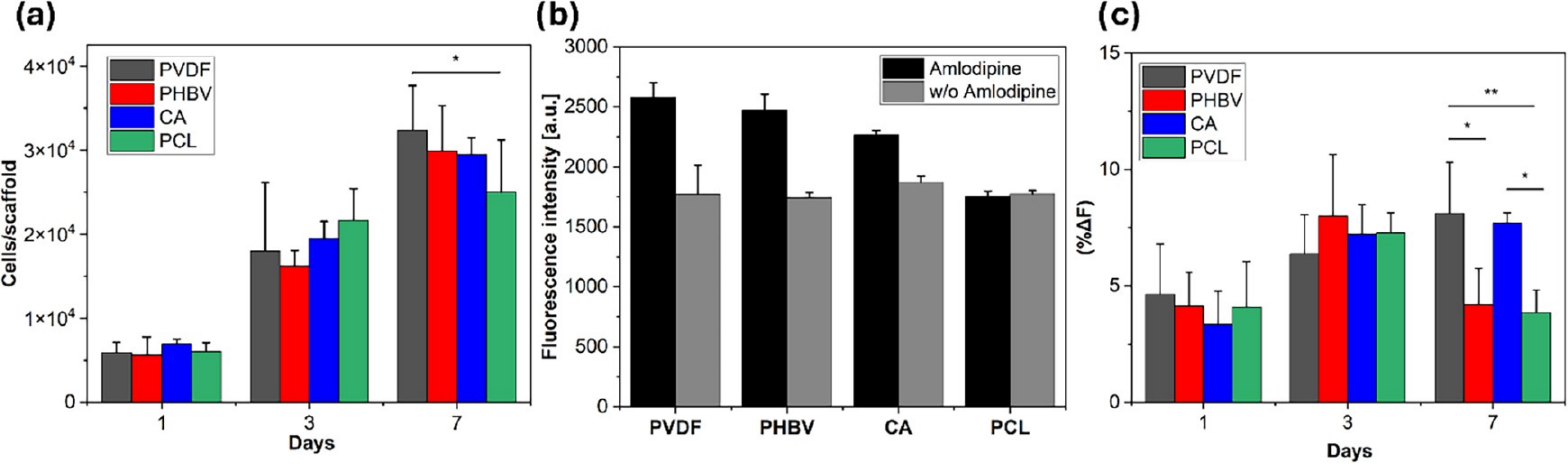
Functional assays of B35 neuronal cells cultured on aligned electrospun fibers. (a) Proliferation of B35 cells on aligned fibers. (b) Intracellular Ca^2+^ levels measured by the Fluo-4 NW assay with and without amlodipine (*p < 0.05). (c) Percent relative fluorescence change (Δ*F*) measured by kinetic analysis after KCl depolarization. %Δ*F* were calculated by (*F*_max_ – *F*_0_)/ *F*_0_ × 100 (*p < 0,05; **p < 0,01).

As shown in Figure 2b, the piezoelectric fibers significantly increased Ca^2+^ influx in the amlodipine-blocked cell group. To determine how piezoelectric scaffolds alter intracellular Ca^2+^ responses, 50 mM KCl was used as an external stimulus. The 50 mM KCl dose corresponded approximately to the EC_50_ region on the dose–response curve. Figure 2c shows the percent relative fluorescence change (%Δ*F*) by kinetic measurements. While no difference was observed on days 1 and 3 in different polymer-based scaffolds, significant differences were observed on day 7. PVDF and CA induced the highest Ca^2+^ response in cells, while PCL produced the lowest response. The increase in Ca^2+^ release on piezoelectric fibers serves as evidence that the piezoelectric effect is altering cellular Ca^2+^ dynamics and modulating Ca^2+^ signaling pathways effectively. It is important to note that this stimulative effect of culture on piezoelectric fibers becomes effective after 3 days, possibly due to the help of piezoelectric stimulation to neuronal maturation.^41,42^ Mature neurons are shown to be more prone to depolarization by elevated external K levels.^43^ Neurite length analysis on electrospun fibers has shown that there is a significant increase in neurite length on PVDF and PHBV fibers (Figure 3a–e). The average neurite lengths were 43.7 ± 15.2 μm, 36.9 ± 19.2 μm, 18.4 ± 14.9 μm, and 24.8 ± 6.8 μm for PVDF, PHBV, CA, and PCL fibers, respectively.

**Figure 3:**
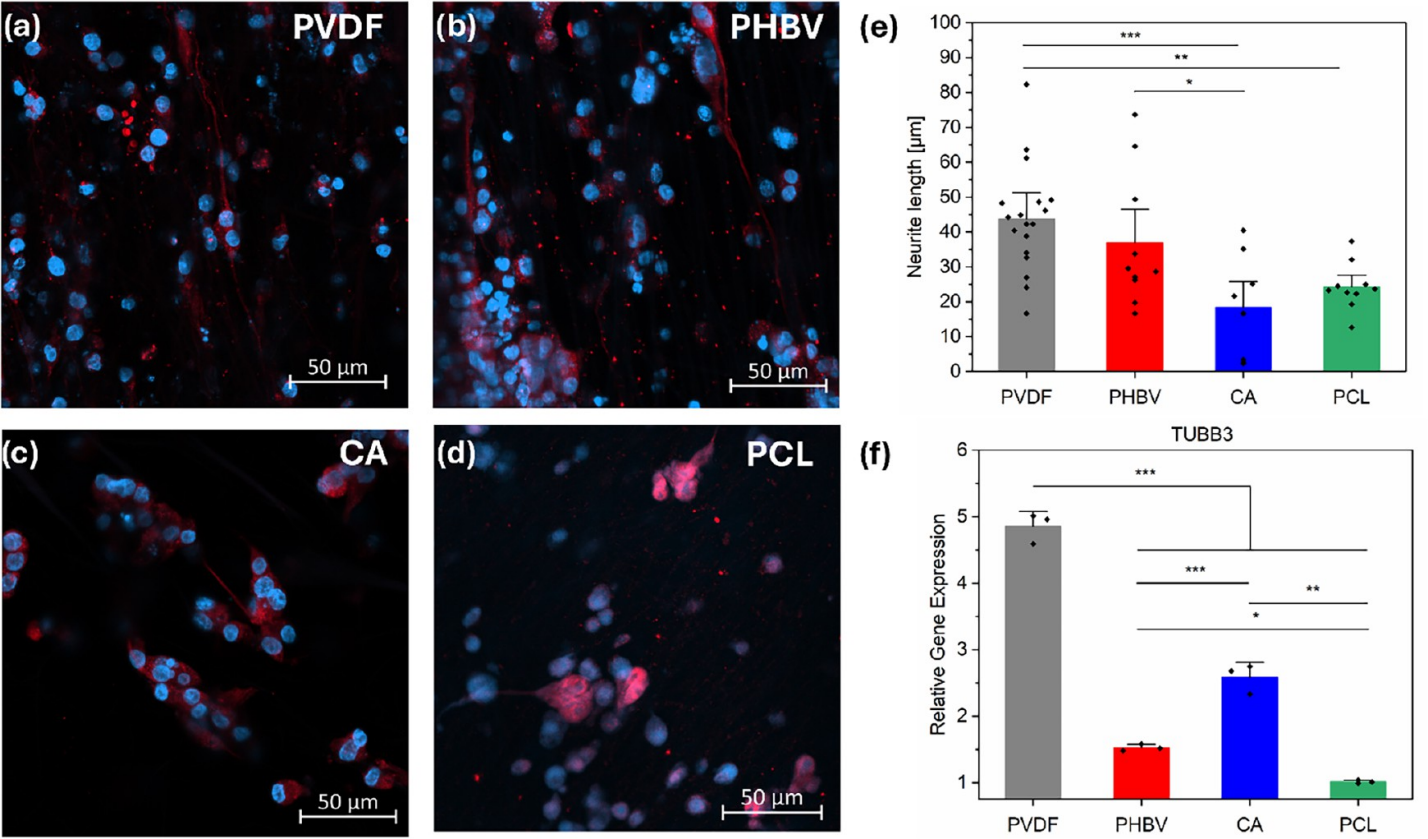
CLSM images of B35 cells growing on aligned electrospun fibers: (a) PVDF, (b) PHBV, (c) CA, (d) PCL (immunofluorescent staining of β3-tubulin with Alexa Fluor 647 and nucleus stained with DAPI); (e) average neurite length, and (f) expression of the TUBB3 gene (*p < 0.05, **p < 0.01, ***p < 0.001).

The expression of β3-tubulin, an early neuronal marker found almost exclusively in neuronal and testicular cells, which plays an important role in neurite growth, was significantly increased on all piezoelectric materials (PVDF, PHBV, CA) compared to PCL. β3-Tubulin (TUBB3) is a critical component of microtubules, which are essential for neurite outgrowth and axonal transport. Increased expression of TUBB3 reflects the formation and elongation of neurites, as microtubules provide the structural framework for these processes.^44^ All scaffolds showed some expression of this early neuronal marker due to the aligned morphology of the scaffolds, which initiated neuronal differentiation of the cells (Figure 3f).

To examine how scaffold morphology and piezoelectric properties influence neuronal behavior, the morphology of B35 cells cultured on electrospun fibers was analyzed by SEM after 7 days of incubation (Figure 4). From the images, we can observe that the cells are growing in local groups shaping along the fibers but also across them (Figure 4a,c,e,g). Especially, larger fibers with sizes above 1 μm facilitate the formation of cellular extensions that can develop into neurites and bridge the gaps between neighboring fibers (Figure 4b,d,f,h). On PVDF, PHBV, and PCL fibers, cells formed clusters, maintained a bulbous morphology, and exhibited distinct neurite extensions along and across the fibers. In contrast, on CA fibers, cells appeared flatter and more widely spread over the surface, resembling growth on 2D substrates. Small CA fibers with low piezoelectric properties do not provide enough stimulation for neural cells to form neurites, as was earlier shown based on CLSM imaging (Figure 3). We can summarize that nerve growth, proliferation, elongation, and neurite formation are regulated by piezoelectric properties of PVDF fibers, but aligned fiber morphology and diameters of about 1 μm and larger also have a significant role in neurite extension, as in the case of PHBV.

**Figure 4:**
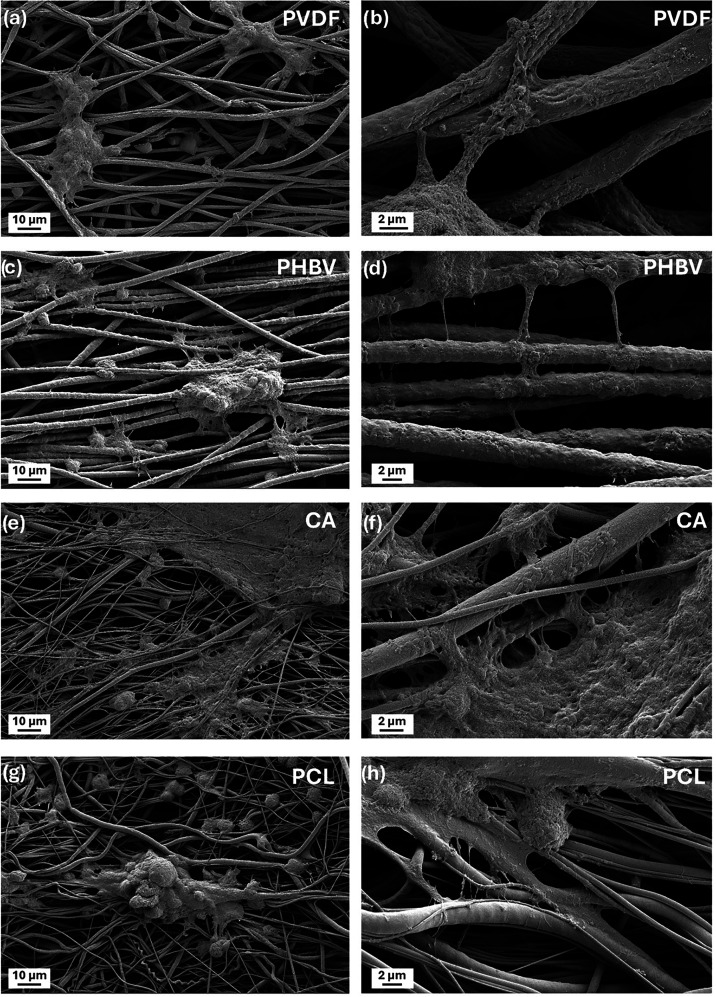
SEM micrographs of neuronal cells growth and cellular protrusions formation on aligned fibers: (a, b) PVDF, (c, d) PHBV, (e, f) CA, (g, h) PCL.

To further investigate the influence of electrospun scaffolds on neuronal survival and apoptosis, the expression levels of key regulatory genes from the BCL-2 family, known for their role in controlling apoptotic processes and widely studied in neuronal cell cultures, were analyzed in B35 cells grown on different fiber types. The mRNA expression levels of the BAX and BCL-2 genes changed after induction on all tested electrospun fibers (Figure 5a,b). Different amounts of downregulation of BAX mRNA expression and upregulation of BCL-2 mRNA expression caused differences in the BAX/BCL-2 ratio on the fibers (Figure 5c). When comparing PCL and CA fibers, no statistically significant change in the BAX/BCL-2 ratio was observed. In contrast, PHBV and PVDF fibers led to a significant decrease in apoptosis inducibility. Aiming to elucidate the molecular mechanisms underlying the neuronal responses observed on the electrospun scaffolds, the expression of genes involved in the WNT/GSK3β signaling pathway, known to regulate neuronal differentiation, neurite outgrowth, and cell survival, was analyzed. Our qPCR analysis revealed higher expression of GSK3-β and WNT on the PHBV and PVDF fibers compared with the other groups (Figure 5d). The higher WNT expression compared to GSK3-β suggests that these scaffolds promote axon elongation. Very little GSK or WNT expression was observed on PCL and CA fibers (Figure 5d).

**Figure 5:**
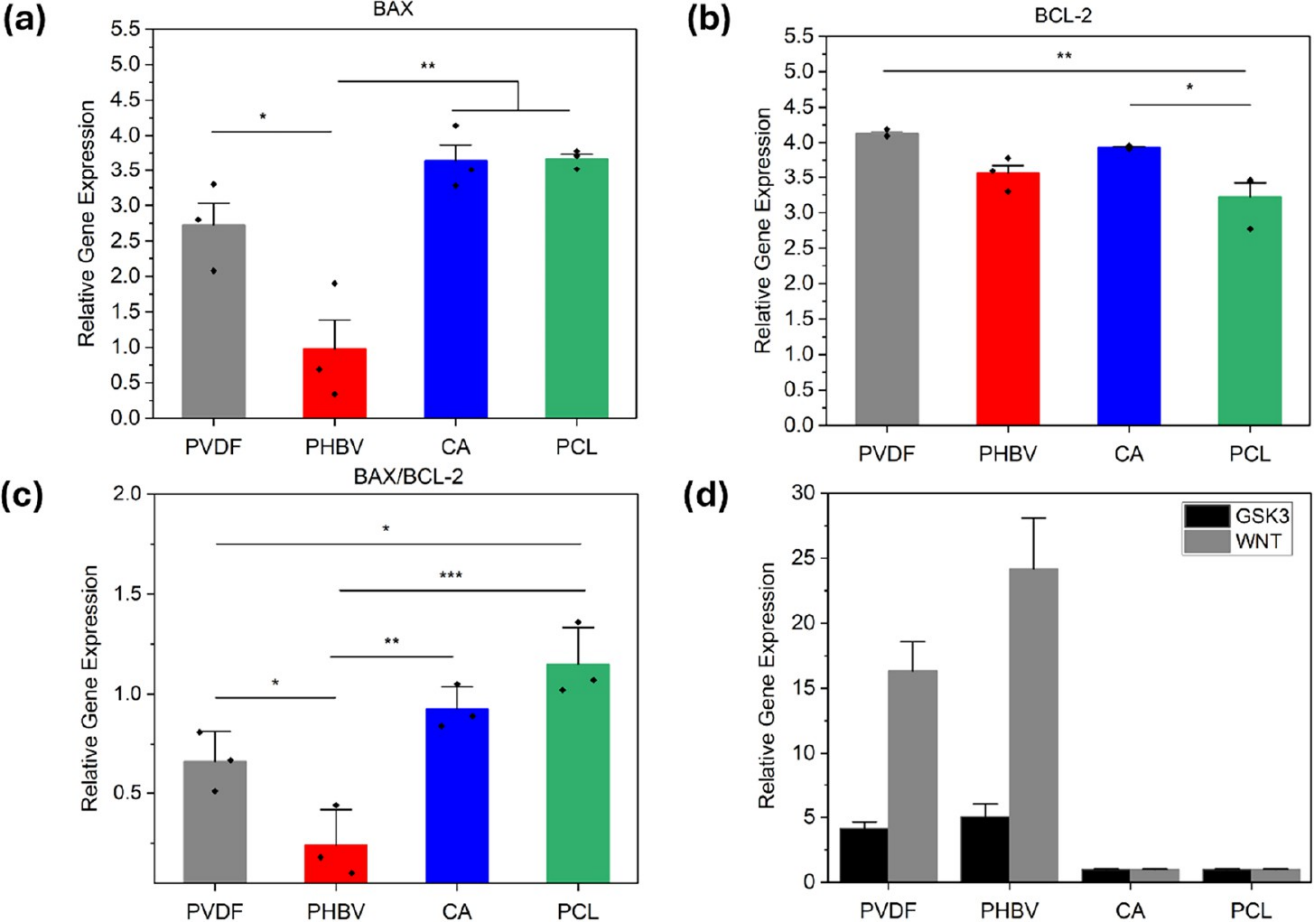
Gene expression associated with cell survival and WNT/GSK3 signaling. Relative mRNA expression of (a) pro-apoptotic (BAX), (b) antiapoptotic (BCL2) genes, (c) BAX/BCL2 ratio, and (d) WNT and GSK3, (*p < 0.05, **p < 0.01, ***p < 0.001).

## 4 Discussion

Manufacturing of fibers with high rotation of the collecting drum during electrospinning causes increased polymer jet stretching, resulting in higher orientation of the molecules along the fiber axis as well as reduced fiber diameters compared to random fibers.^45^ This reduction of fiber diameter with high rotation during electrospinning was also observed in the current study when compared with literature data for random fibers. Aligned PVDF fibers were thinner by 32%,^32^ PHBV fibers by 17%,^30^ and CA fibers by 7%^25^ with respect to random fibers (Figure 1e). Additionally, in aligned fibers, the pore sizes are reduced, and their shapes become more elongated, resulting in a more densely packed scaffold structure.^46^ The morphological changes and molecular orientation within individual fibers during aligned fiber manufacturing affect their surface ζ-potential. Marques-Almeida et al. demonstrated that neurons cultured on negatively charged PVDF films produced by solvent casting and subsequent poling exhibited enhanced metabolic activity, accelerated maturation, stronger βIII-tubulin signals, and increased expression of neuron-specific marker genes.^47^ Biomaterials’ surface potential, which corresponds to the accumulation of positive or negative charges, is one of the elements guiding cellular processes by electrostatic interactions with ions, amino acids, and proteins present in culture media.^48^ Adhesion of right biomolecules onto biomaterials’ surface is therefore a mediating factor in cell attachment and further tissue development.^49^ Another property considered to affect protein adsorption on biomaterials’ surface and, therefore, regulating neural processes is wettability.^50^ Regardless of hydrophobic properties, fibrous scaffolds provide beneficial structural support and instructive microenvironment for many tissue types, including neurons, guiding their attachment, development, and regeneration.^51,52^

While the effects of fiber alignment, density, and diameter on neuronal viability and neurite length have been extensively studied, studies on the effects of fiber surface nanotopography on cells are limited.^53,54^ A study by Lee et al. examined the in vitro effects of surface nanotopography on PC12 cells and primary rat hippocampal neurons.^53^ It has been reported that the surface protrusions of polypyrrole-coated electrospun poly­(lactic-co-glycolic acid) fibers did not significantly affect neurite extension in PC12 cells or primary hippocampal neurons. In another study, embryonic chick DRGs were cultured on electrospun fibers with smooth surface protrusions and depressions. This study also showed that DRGs on fibers with pits and depressions extended approximately 65% more neurites than DRGs cultured on smooth fibers.^54^ The presented study also shows that rough fibers (PHBV and PVDF) have significantly higher diameters compared with the smoother fibers (CA and PCL), Figure 1a–d. In the study conducted by Ziemba et al., similar to our study, the average diameter of fibers with pits and depressions was larger than that of smooth fibers.^54^ In our research, fibers with surface nanotopography and larger diameters (PVDF and PHBV) strongly supported the neurite growth and elongation compared to smooth CA and PCL fibers, as shown in Figure 1e. Therefore, the effects of electrospun fiber surface nanotopography on neurons are consistent. Another study examining the effects of electrospun fiber surface nanotopography on neurites showed that nanotopographies can increase overall neurite growth and branching.^55^ Overall, these findings indicate that surface nanotopography, along with a larger fiber diameter, plays a key role in promoting neuronal adhesion, neurite outgrowth, and elongation. This highlights the importance of a controlled surface morphology in designing electrospun scaffolds for neural tissue engineering.

The use of piezoelectric materials as scaffolds in tissue engineering provides electrical stimulation without the need for electrodes, external power sources, or implantable batteries. Piezoelectric scaffolds can generate electrical pulses as a result of cell attachment and migration or due to transient deformations caused by body movements. These electrical charges interact with voltage-gated calcium channels (VGCCs) on the neuronal membrane, promoting the influx of calcium ions into the cell.^8^ This calcium influx plays a crucial role in several cellular processes, such as synaptic transmission, gene expression, and plasticity.^37,56^ A key marker of synaptic transmission is Ca^2+^ metabolism, which triggers synaptic vesicle exocytosis and initiates neurotransmitter release. Direct electrical stimulation has been shown to regulate VGCCs and affect cellular processes.^57,58^ Piezoelectric fibrous scaffolds have been proven to contribute to neurite length elongation in many studies.^59,60^ While fibers provide topographic cues to cells, piezoelectric properties also provide electrical stimulation.^59^ In a study with PHBV/Collagen fibers, PC12 cells cultured on aligned PHBV/collagen nanofibers oriented along the fiber direction exhibited elongated morphologies with bipolar neurite extensions.^61^ In our study, CA did not significantly contribute to neurite outgrowth. Neurite elongation was highest on PHBV and PVDF fibers, Figure 3e. Previously, it was shown that with the application of ultrasound on piezoelectric PVDF membranes, PC12 cells grew longer neurites.^62^ In another work, the effect of PVDF on neurite elongation was studied with a primary neuron culture. The fibers helped dorsal root ganglion (DRG) neurons to grow longer neurites, and the average neurite length was up to 2500 μm.^60^ In another study with human neural stem cells, human neural stem/progenitor cells were seeded on PVDF membranes and their contribution to neurite elongation was shown.^38^

It has been shown in the literature that aligned fibers help the maturation of neural cells through neurite anchorage and subsequent elongation parallel to the fiber axis by bridging the cells to the fibers with an increased number of focal contacts.^63–67^ Lim et al. had suggested that the fiber topography was adequate by itself to promote canonical WNT pathway activation that regulates neurite elongation.^68^ For PCL and PHBV, neurite length is increased despite low β3-tubulin expression levels. It can be considered that on these fibers neurite elongation is associated with the activation of alternative actin dynamics. This can also indicate weak microtubule activity and fragile neurite formation.^69^ In particular, the low piezoelectric properties of PCL (Figure 1h) do not create mechanical tension on the cells, which may promote alternative elongation pathways dependent on actin enrichment, thereby supporting neurite outgrowth independently of microtubules. In CA, although β3-tubulin is highly expressed, neurite lengths appear to be short. This observation, in line with studies showing that β3-tubulin expression begins in the early stages of neuronal differentiation, whereas neurite outgrowth occurs later,^70,71^ suggests that the cells are still in an early phase of maturation at day 7. The moderate piezoelectric properties of CA may provide a certain degree of mechanical stimulation to the cells, potentially triggering differentiation; however, it might not have yet offered sufficient environmental support for fully mature neurite formation. In PVDF, the simultaneous high expression of β3-tubulin and increased neurite length (Figure 3e,f) indicate the establishment of an ideal environment for neuronal differentiation. This suggests that the cells are at a more advanced stage of maturation.^72,73^ These findings demonstrate that the high piezoelectric capacity of PVDF promotes neuronal differentiation and mature neurite outgrowth. The continuous electrical microstimuli provided by PVDF appear to support microtubule stabilization, thereby contributing to the development of mature and functional neuronal morphology.^74–76^

It is noteworthy that although the cells exhibited neurite extensions on the PHBV fibers comparable to PVDF, this morphological maturity was not supported by β3-tubulin expression and a stimulus-dependent intracellular Ca^2+^ response. It has been previously reported that increased neurite length does not always correlate with functional maturity, particularly as β3-tubulin post-translational modifications, which determine microtubule stability, and mitochondrial Ca^2+^ regulation are known to play a critical role in functional differentiation.^77,78^ As both neurite length and β3-tubulin expression, as well as the Ca^2+^ response, were at their highest levels on the PVDF, it is suggestive that PVDF supports functional maturation more strongly not only through morphological changes but also through ion channel functions and cytoskeletal organization.

The BAX/BCL-2 ratio is believed to be an essential determinant of apoptotic tendencies.^79–83^ The BAX/BCL-2 ratio was shown to reach a maximum level when neuronal death rates are high both in vivo and in vitro, showing a pro-apoptotic tendency.^84^ It is thought that BCL-2 counteracts the pro-apoptotic function of BAX by preventing its oligomerization.^85,86^ Although BCL-2′s neuroprotective effects are well-established,^87,88^ some studies have shown its involvement also in neuronal development and differentiation.^89,90^ Depletion of BCL-XL, a member of the BCL-2 family, has been shown to cause neurite growth arrest and reduced neurite length, indicating an impaired ability to form a normal neuronal network in hippocampal neurons.^91^ Similarly, overexpression of BCL-2 was shown to enhance Ca^2+^ signaling, promote neurite growth in central nervous system neurons^92,93^ and differentiated PC12 cells,^94^ while inhibition of BCL-2 was shown to induce delayed differentiation of human neural progenitor cells.^95^ It is also thought that BCL-2 enhances mitochondrial ATP production to meet the high energy demand of neurons and supports synaptic transmission.^96,97^ Increased BCL-2 expression (reduced BAX/BCL-2 ratio) on PHBV and PVDF fibers (Figure 5a–c) is consistent with increased neurite growth and improved neuroregenerative capacity on these fibers. Although β3-tubulin expression and Ca^2+^ response show a more favorable effect of PVDF fibers over PHBV fibers on neurite elongation and neuronal maturation, PHBV fibers create a more pronounced antiapoptotic and therefore more neuroprotective effect on B35 cells.

WNTs are a family of lipid-modified glycoproteins that act as secreted signaling molecules regulating crucial cell-to-cell communication during development and in adult tissues.^98^ They bind to specific receptors and control key processes, such as cell fate determination, tissue patterning, polarity, proliferation, maturation, and motility. Glycogen synthase kinase 3 β (GSK3-β), an essential part of this pathway, is a serine/threonine kinase that plays a vital role in various cellular processes, including neuronal development and plasticity. It is also known that WNT/GSK3 signaling regulates not only β-catenin-mediated gene expression, which is involved in neurogenesis,^99,100^ but also mitochondrial balance in neuronal maturation. Also, WNT activation with GSK3 inhibition increases BCL-2 expression while suppressing BAX levels, thereby supporting cell survival and neuronal differentiation.^101,102^

WNT signaling plays a multifaceted role in the regulation of neurite outgrowth. Upregulation of WNT ligands and activation of the WNT/β-catenin pathway have been shown to promote neurite outgrowth and neuronal differentiation.^103^ This activation leads to increased expression of genes encoding cytoskeletal proteins such as actin and tubulin, cell adhesion molecules, and growth factors that support neurite elongation and branching.^104^ GSK3 plays an important role in the control of neuronal polarization by cross-talk with other signaling pathways.^105,106^ It is present in growing axons but is removed from axons at the end of axonogenesis, being restricted to neuronal cell bodies and dendrites in adults.^107^ In a previous study with primary chicken sensory neurons, GSK3-β inhibition was found to reduce axon elongation but increase the area of the growth cone.^108^ It reversibly reduces axon growth rate, enlarges axonal growth cones and induces spreading areas along neurite shafts.^107^ When WNT signaling is activated, GSK3-β activity is inhibited, and it cannot phosphorylate β-catenin, and β-catenin accumulates in the cytoplasm and translocates to the nucleus, promoting cell differentiation.^103,109,110^ We can simply say that GSK3-β promotes the formation and propagation of axonal growth cones, suppresses axonal elongation through a canonical signaling transduction pathway, and facilitates axonal regeneration and activation of neurotrophins.^111–113^ In contrast, WNT is associated with axonal elongation, cell proliferation, self-renewal, specification, and neural precursor differentiation in the CNS.^113,114^

Our findings suggest that PVDF contributes to functional maturation by activating the WNT/β-catenin pathway via increased intracellular Ca^2+^ response. The literature reports that Ca^2+^ signals are an important trigger of the Wnt/Ca^2+^ pathway and that this pathway regulates both neuronal differentiation and cell survival via GSK3-β inhibition.^101^ GSK3-β inhibition increases antiapoptotic BCL-2 expression while suppressing pro-apoptotic BAX levels, thereby supporting mitochondrial stability and functional maturation.^102^ Our findings suggest that PVDF positively regulates this axis via Ca^2+^-mediated WNT activation due to its piezoelectric properties. Electrical activity has also been shown to directly modulate the BAX/BCL-2 ratio.^115^ Therefore, our results reflect this modulation, as the two polymers having the highest piezoelectricity (PVDF and PHBV) induce the lowest BAX/BCL-2 ratio. Since PHBV has lower piezoelectricity but also a lower BAX/BCL-2 ratio, it implies that PHBV has other neuroprotective properties that further decrease the apoptotic tendency. It was shown before that 3-hydroxybutyrate and its derivatives had an antiapoptotic effect on neural and glial cells.^116^ Xiao et al. suggested that this effect may be due to the regulation of Ca^2+^ influx, but the exact mechanism is yet to be discovered. Overall, these results indicate that the activation of the WNT/GSK3-β signaling pathway, particularly on PVDF and PHBV scaffolds (Figure 5d), contributes to enhanced neuronal differentiation, reduced apoptosis, and functional maturation, likely mediated by the piezoelectric stimulation, microsized aligned morphology of the fibers with nanotopography, and associated Ca^2+^ signaling.

## 5 Conclusions

We successfully cultured B35 neuronal cells on four types of aligned electrospun polymer fibers. Among them, PVDF, PHBV, and CA displayed piezoelectric properties in decreasing order, while PCL served as a nonpiezoelectric control. Controlled fiber alignment produced distinct surface nanotopographies, grooved for PVDF, pitted for PHBV, and smooth for CA and PCL, with smaller diameters and elongated pores that provided directional cues for neurite extension. Despite their hydrophobicity, all scaffolds supported cell adhesion and proliferation over 7 days facilitated by their fibrous architecture and negatively charged surfaces.

Piezoelectric testing showed a clear hierarchy of activity, with PVDF exhibiting the strongest response, PHBV and CA showing moderate activity, and PCL showing negligible activity. This trend corresponded closely to the cell viability, calcium signaling, and neurite elongation. The results indicate that piezoelectricity can activate the WNT/GSK3β pathway and enhance intracellular Ca^2+^ influx, triggering neuronal maturation evidenced by β3-tubulin expression and extensive neurite formation. This pathway was most strongly upregulated on PVDF and PHBV fibers, and notably, the piezoelectric environment reduced the inhibitory effect of amlodipine on voltage-gated Ca^2+^ channels after a prolonged culture. Importantly, our findings confirm that piezoelectricity alone does not fully explain neuronal maturation. Fiber alignment, diameter, and surface nanotopography also contribute significantly to neuronal behavior by modulating Ca^2+^ dynamics and cytoskeletal organization. Among the tested materials, PHBV emerged as a promising alternative to PVDF, offering moderate piezoelectric activity, the lowest pro-apoptotic BAX/BCL-2 ratio, and strong neuroprotective effects. Considering that PVDF is increasingly restricted due to fluoropolymer regulations, PHBV provides a sustainable, regulation-compliant substitute for neural tissue engineering applications.

Overall, the data demonstrate that nanoscale alignment, surface charge, and piezoelectric polarization act cooperatively to enhance the Ca^2+^ influx, neurite development, and cell survival. PVDF remains a robust choice for studying piezoelectric neurostimulation, while PHBV represents a practical and biocompatible route toward new neural scaffolds and regenerative therapies conforming to EU regulations.

## Data Availability

Data will be made available on request.
